# An autoradiographic evaluation of AV-1451 Tau PET in dementia

**DOI:** 10.1186/s40478-016-0315-6

**Published:** 2016-06-13

**Authors:** Val J. Lowe, Geoffry Curran, Ping Fang, Amanda M. Liesinger, Keith A. Josephs, Joseph E. Parisi, Kejal Kantarci, Bradley F. Boeve, Mukesh K. Pandey, Tyler Bruinsma, David S. Knopman, David T. Jones, Leonard Petrucelli, Casey N. Cook, Neill R. Graff-Radford, Dennis W. Dickson, Ronald C. Petersen, Clifford R. Jack, Melissa E. Murray

**Affiliations:** Department of Radiology, Mayo Clinic, Rochester, MN USA; Department of Neurology, Mayo Clinic, Rochester, MN USA; Department of Anatomic Pathology, Mayo Clinic, Rochester, MN USA; Department of Neuroscience, Mayo Clinic, Jacksonville, Florida USA; Department of Neurology, Mayo Clinic, Jacksonville, Florida USA

**Keywords:** AV-1451, Tau, Alzheimer’s disease, TDP-43, Pick Disease, Corticobasal degeneration, Progressive supranuclear palsy, Tauopathy, Pick’s disease, Atypical Alzheimer’s disease, Frontotemporal dementia

## Abstract

**Background:**

It is essential to determine the specificity of AV-1451 PET for tau in brain imaging by using pathological comparisons. We performed autoradiography in autopsy-confirmed Alzheimer disease and other neurodegenerative disorders to evaluate the specificity of AV-1451 binding for tau aggregates.

**Methods:**

Tissue samples were selected that had a variety of dementia-related neuropathologies including Alzheimer disease, primary age-related tauopathy, tangle predominant dementia, non-Alzheimer disease tauopathies, frontotemporal dementia, parkinsonism, Lewy body disease and multiple system atrophy (*n* = 38). Brain tissue sections were stained for tau, TAR DNA-binding protein-43, and α-synuclein and compared to AV-1451 autoradiography on adjacent sections.

**Results:**

AV-1451 preferentially localized to neurofibrillary tangles, with less binding to areas enriched in neuritic pathology and less mature tau. The strength of AV-1451 binding with respect to tau isoforms in various neurodegenerative disorders was: 3R + 4R tau (e.g., AD) > 3R tau (e.g., Pick disease) or 4R tau. Only minimal binding of AV-1451 to TAR DNA-binding protein-43 positive regions was detected. No binding of AV-1451 to α-synuclein was detected. “Off-target” binding was seen in vessels, iron-associated regions, substantia nigra, calcifications in the choroid plexus, and leptomeningeal melanin.

**Conclusions:**

Reduced AV-1451 binding in neuritic pathology compared to neurofibrillary tangles suggests that the maturity of tau pathology may affect AV-1451 binding and suggests complexity in AV-1451 binding. Poor association of AV-1451 with tauopathies that have preferential accumulation of either 4R tau or 3R tau suggests limited clinical utility in detecting these pathologies. In contrast, for disorders associated with 3R + 4R tau, such as Alzheimer disease, AV-1451 binds tau avidly but does not completely reflect the early stage tau progression suggested by Braak neurofibrillary tangle staging. AV-1451 binding to TAR DNA-binding protein-43 or TAR DNA-binding protein-43 positive regions can be weakly positive. Clinical use of AV-1451 will require a familiarity with distinct types of “off-target” binding.

**Electronic supplementary material:**

The online version of this article (doi:10.1186/s40478-016-0315-6) contains supplementary material, which is available to authorized users.

## Introduction

Tauopathies constitute a group of neurodegenerative disorders associated with molecular alterations in the microtubule associated protein tau that can be sub-classified by the predominant species of tau that accumulates within neurons and glia [1]. Tau protein promotes tubulin polymerization and acts to stabilize microtubules, which are abundant in axons and important for many neuronal processes, such as axoplasmic transport. Extensive post-translational modifications of tau (e.g., phosphorylation) affect its functional properties, leading to dissociation from microtubules and abnormal accumulation in intracellular inclusions in neurons known as neurofibrillary tangles (NFT). Neuronal accumulation of abnormal tau and its attendant neuronal loss correlate with cognitive deficits in AD [2, 3]. The relative amounts of neuronal and glial accumulation and the neuroanatomical distribution of tau define a range of neurodegenerative tauopathies, including tangle predominant dementia [4, 5], argyrophilic grain disease (AGD) [6], Pick disease (PiD) [7], progressive supranuclear palsy (PSP) [8] and corticobasal degeneration (CBD) [9]. The tau gene (*MAPT*) has 13 exons, and it undergoes alternative splicing of exons 2, 3 and 10 to generate 6 isoforms [10]. Exon 10 encodes one of four 32-amino acid conserved sequences in the microtubule-binding domain of tau. Tau that includes exon 10 has four repeats (4R tau) in the microtubule binding domain, while tau that excludes exon 10 has three repeats (3R tau) [1]. Variable amino terminal inserts (N) are also present leading to 6 isoforms denoted as 2N4R, 2N3R, 1N4R, 1N3R, 0N4R, and 0N3R, the largest of which being 2N4R with 441 amino acids [1]. The composition of fibrillar tau also differs across tauopathies, related to stoichiometry of the insoluble isoforms that accumulate in the brain, levels of hyperphosphorylation, and the morphology of the fibrillar aggregates (paired helical filaments (PHF) or straight filaments (SF), (reviewed in [11]). Although the specific nature of tau deposits and their neuroanatomical distributions have guided neuropathologic diagnosis of tauopathies, diagnostic accuracy during life remains poor in many of these disorders. In vivo imaging of tau pathology may greatly improve not only diagnostic accuracy, but enhance efforts toward early detection and treatment.

**Fig. 1:**
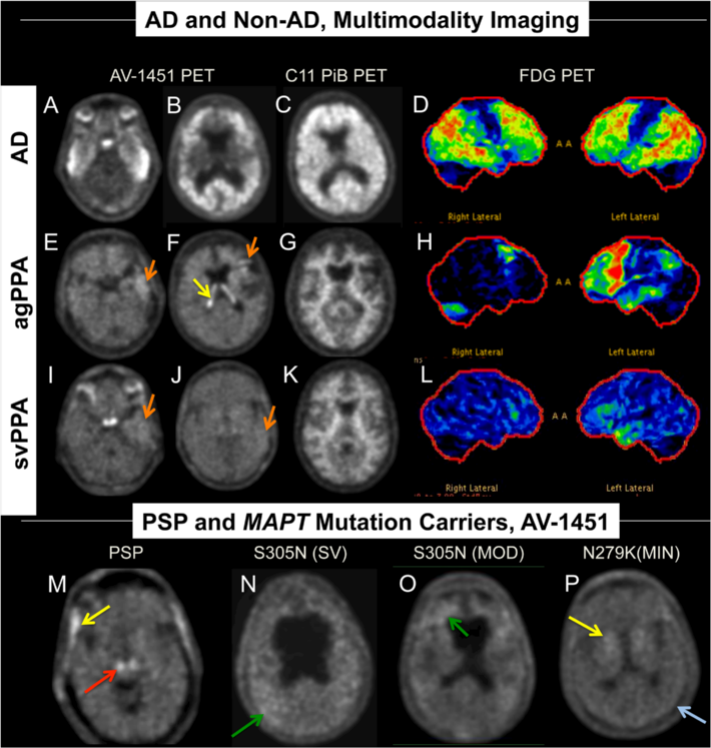
Examples of AV-1451 PET scans in subjects with various diseases. Correlative PET imaging (AD and Non-AD, Multimodality Imaging, rows 1-3) in an AD subject (1) with high AV-1451 signal (*white regions*), high PiB signal (*white regions*) and reduced FDG signal in an “AD” pattern (temporal, parietal and some frontal; colors indicate hypometabolism); an agPPA subject (2) with mild signal in the left temporal and frontal lobes on AV-1451 PET (*orange arrows*), normal PiB signal, and an “agPPA pattern” on FDG showing left sided dominant frontal and temporal hypometabolism and a svPPA subject (3) with very mild signal in the left temporal lobe on AV-1451 PET (*orange arrows*), normal PiB signal, and an svPPA pattern on FDG showing left sided dominant temporal hypometabolism. On row 4 (PSP and MAPT Mutation Carriers, AV-1451), from left to right, are AV-1451 PET images showing scattered, minimal signal in tauopathies secondary to PSP and *MAPT* mutations. The PSP subject showed signal in the brainstem that is likely nonspecific (*red arrow*). The left *MAPT* S305N mutation subject has severe (SV) symptoms and secondary ventricular enlargement and the next to the right has moderate (MOD) symptoms. The *MAPT* N279K mutation subject on the far right has relatively minimal (MIN) symptoms. Some very mildly increased AV-1451 signal is identified in the *MAPT* S305N subjects diffusely (*green arrows*) but it is clearly less intense than in the AD subject (1) but only mildly more than in the *MAPT* N279K subject (*blue arrow*). *Yellow arrows* show meningeal (PSP subject), basal ganglia (*MAPT* N279K subject), and choroid plexus signal (agPPA subject) and are likely non-specific binding regions

Over the last decade, amyloid imaging using positron emission tomography (PET) scanning has successfully demonstrated evidence of cerebral Aβ deposition in individuals with AD [12] and longitudinal amyloid imaging of neurologically normal individuals shows transition to amyloid positive status in those at risk for AD [13], such that determinations of amyloid status is being integrated into clinical diagnosis of AD [14]. Since NFTs correlate with cognitive decline in AD better than amyloid [15, 16], it is important to better understand the status of tau deposition. As such, recent efforts have focused on developing tau-imaging agents. PET imaging with AV-1451 has shown highly specific binding affinity to tau in AD patients [17, 18]. However, understanding AV-1451 binding to tau in AD, atypical AD and non-AD tauopathies and its relative binding to other pathologic aggregates is also of great importance and is beginning to be elucidated [19]. In addition, anecdotal reports of purported “off-target” binding of AV-1451 have been reported in diseases not considered to be associated with tau accumulation (unpublished data). In patients at our institution, PET imaging with AV-1451 has shown AV-1451 binding prominently in AD, but also in “off-target” sites, and minimally in clinically diagnosed frontotemporal dementia (FTD) and non-AD tauopathies (unpublished data) (Fig. 1). Understanding the relationship between AV-1451 binding and pathology could help define the potential utility of this technology, improve our understanding of the pathologic mechanisms of dementia and direct targeted therapeutic approaches. It is essential, therefore, to determine the specificity of AV-1451 PET and its pathologic correlates. Using autoradiography and immunohistochemistry, our study had three main objectives: 1) to evaluate the binding properties of AV-1451 to AD-tau and compare it to several non-AD tauopathies; 2) to evaluate the specificity of AV-1451 binding in disorders not associated with significant tau deposition such as TDP-43 proteinopathies and α-synucleinopathies; and 3) to investigate the nature and patterns of “off-target” AV-1451 binding.

## Materials and methods

### Case selection

Autopsy cases were selected with a range of neurodegenerative disorders with and without tau deposition from the brain bank for neurodegenerative disorders at Mayo Clinic in Jacksonville, Florida. All work in this paper has been approved by the Mayo Foundation and have been performed in accordance with the ethical standards as laid down in the 1964 Declaration of Helsinki and its later amendments or comparable ethical standards. To examine binding specificity of AV-1451 to tau we included neuropathologically normal individuals with no NFT or Aβ deposits (*n* = 3), neuropathologically normal individuals with minimal NFT and substantial Aβ deposits ((“pathological aging” (PA), *n* = 2), neuropathologically normal individuals with NFT and no Aβ deposits (also known as primary age-related tauopathy (PART) [20], (*n* = 3), tangle predominant dementia (*n* = 3), and AD (*n* = 3) [21, 22]. We also examined non-AD tauopathies that included PiD (*n* = 3) [23], CBD (*n* = 2) [9], PSP (*n* = 3) [8], AGD (*n* = 2) [24], and frontotemporal dementia and parkinsonism linked to chromosome 17 due to P301L, N279K, or R406W mutations in *MAPT* (FTDP-17, *n* = 5) [23, 25]. We examined other non-tauopathies that included frontotemporal lobar degeneration with TDP-43 immunoreactive lesions (FTLD-TDP, *n* = 4) [23], Lewy body disease (LBD, *n* = 2) [26] and multiple system atrophy (MSA, *n* = 1) [27]. We assessed “off-target” binding in all of these cases, To assess “off-target” binding of AV-1451 to iron-associated deposits, a patient with neurodegeneration with brain iron accumulation [NBIA] and a patient with superficial siderosis associated with chronic subarachnoid hemorrhages were studied [28].

### Clinically relevant questions of interest

Autoradiographic binding and immunohistochemical evaluation of these 38 cases and controls were used to investigate several clinically relevant questions of interest as described below. Some cases and controls were used for more than one question of interest when applicable (Tables 1 and 2).

1. Tau maturity, Atypical AD and AD
  Tissue from 13 cases was used to assess the binding of AV-1451 in cases with progressive increases in Braak NFT stages [29]. Select cases with tangle predominant dementia or PART with minimal or no Aβ deposits were compared to AD with extensive Aβ deposits. Cases included were Braak Stage IV or less and Thal amyloid phase of 0 (*n* = 6), Braak Stage IV or less and Thal amyloid phase of 2 - 3 (*n* = 2), and advanced AD with Braak VI and Thal phase 5 (*n* = 2). Normal controls with neither tau nor Aβ deposits (*n* = 3) were also included for comparison.
2. Isoform variations of tau and AV-1451
  Tissue from 18 cases was used to compare and contrast binding of AV-1451 in 3R + 4R, 3R, and 4R predominant tauopathies. Of the three AD cases, one was classified as hippocampal sparing AD based upon the relative abundance of tangles in the cortex compared to hippocampus [30]. An R406W *MAPT* mutation carrier, which are known to exhibit both 3R + 4R tau pathology, was also included to investigate binding [25]. Cases with pathologically confirmed PiD were chosen to represent 3R tauopathies, while CBD, PSP, AGD and FTDP-17 (two each for N279K [31] and P301L [32] *MAPT* mutations) were chosen to represent 4R tauopathies.
3. TDP-43 and AV-1451
  Brain tissue from 9 selected cases was used to address the issue of possible binding of AV-1451 to TDP-43 pathology. Anecdotal reports of in-vivo AV-1451 PET uptake in semantic dementia variants of primary progressive aphasia (svPPA), an entity that is typically associated with FTLD-TDP pathology [33], has raised this as an issue of interest (Fig. 1). Autopsied cases with FTLD-TDP pathology and contrasting cases with clinically diagnosed svPPA but with Tau pathology at autopsy were selected as cases for this comparison.
4. AV-1451 and α-synuclein
  Tissue from 4 cases was used to assess any potential binding of AV-1451 by α-synuclein pathology. Cases with pathologically confirmed LBD with widespread neocortical involvement (i.e. diffuse LBD, *n* = 2) and MSA (*n* = 1) were selected for this aim.
5. AV-1451 “Off-target Binding”
  Tissue from several of the above cases was assessed for “off-target” AV-1451 binding. These were chosen based on anecdotal reports of AV-1451 PET (unpublished data) and observations from tissue samples in regions that were not expected to have tau pathology, including basal ganglia, choroid plexus, pituitary, vessels, and subpial surface. AV-1451 binding in the midbrain was assessed. Cases with NBIA and superficial siderosis were included to assess the potential of AV-1451 binding to iron deposits.

**Table 1:** Subject characteristics of tauopathies

Case	Clinical diagnosis	Neuropathologic diagnosis	TDP type	Tau isoform	Braak	Thal	Age	Sex	Fig.
Tau staging/maturity									
1	Normal	Normal	0	--	0	0	81	F	
2	Normal	Normal	0	--	0	0	68	M	Fig. 2 A-C; Fig. 8 A-C
3	Normal	Normal	0	--	0	0	65	M	
4	Normal	PA	0	3R + 4R	III	3	94	F	
5	MCI	PA	0	3R + 4R	III	2	88	F	Fig. 2 D-F
6	svPPA	AD (HpSp)/DLBD	0	3R + 4R	VI	5	60	M	Fig. 2 G-I
7	AD	AD/DLBD	0	3R + 4R	VI	5	78	F	Fig. 3 A-D
8	PSP	PART	0	3R + 4R	III-IV	0	57	F	
9	Normal	PART	0	3R + 4R	III-IV	0	89	F	
10	Normal	PART	0	3R + 4R	IV	0	82	F	Fig. 2 J-L
11	MCI	Tangle predominant dementia	0	3R + 4R	III	0	81	F	
12	AD	Tangle predominant dementia	0	3R + 4R	IV	0	82	M	Fig. 2 M-O
13	AD	Tangle predominant dementia/AGD	B	3R + 4R	III-IV	0	80	F	
Tau isoforms									
6	svPPA	AD (HpSp)/DLBD	0	3R + 4R	VI	5	60	M	Fig. 4 A,B
14	svPPA	PiD	0	3R	0	0	74	M	Fig. 4 C,D
15	AD	PiD	0	3R	I	1	70	M	Fig. 4 E-H
16	PiD	PiD	0	3R	0	0	72	M	Fig. 4 I-L
17	agPPA	CBD/PA	0	4R	I-II	2	67	M	Fig. 5 A-D
18	AD	CBD/AGD	0	4R	II	0	67	M	Fig. 5 E,F
19	agPPA	PSP/AGD	0	4R	II	0	80	M	Fig. 5 G-J
20	PSP	PSP	0	4R	II-III	1	68	F	Fig. 5 K,L
21	PSP	PSP	0	4R	I	0	65	M	
22	MCI	AGD	0	4R	III	0	90	M	Fig. 5 M,N
23	Normal	AGD	0	4R	III	1	91	M	Fig. 5 O,P
24	PPND	FTDP-17 (*MAPT* N279K)	0	4R	0	0	49	M	Fig. 6 A-F
25	PPND	FTDP-17 (*MAPT* N279K)	0	4R	0	0	50	F	Fig. 6 G,H
26	FTD	FTDP-17 (*MAPT* P301L)	0	4R	0	0	53	M	Fig. 6 K-N
27	FTD	FTDP-17 (*MAPT* P301L)	0	4R	I-II	0	52	M	Fig. 6 O,P
28	FTD	FTDP-17 (*MAPT* R406W)	0	3R + 4R	5	0	66	F	Fig. 6 Q-T
7	AD	AD/DLBD	0	3R + 4R	VI	5	78	F	

De-identified case numbers are referred to throughout the text and within Figs. TDP-43 type was performed using the recommended harmonized classification system [33]. Braak refers to neurofibrillary tangle stage [29] and Thal refers to amyloid phase [37]. Abbreviations: *MCI* mild cognitive impairment, *svPPA* semantic variant of primary progressive aphasia, *AD* Alzheimer’s disease, *PSP* progressive supranuclear palsy, *PiD* Pick’s disease, *agPPA* agrammatic variant of PPA, *PPND* pallido-ponto-nigral degeneration, *FTD* frontotemporal dementia, *HpSp* hippocampal sparing AD, *DLBD* diffuse Lewy body disease, *AGD* argyrophilic grains disease, *CBD* corticobasal degeneration, *FTDP*-17 frontotemporal dementia and parkinsonism linked to chromosome 17, *3R* 3 *repeat tau*, *4R* 4 repeat tau, *F* female, and *M* male

**Table 2:** Subject characteristics of TDP-43, α-synuclein and off-target binding studies

Case	Clinical diagnosis	Neuropathologic diagnosis	TDP type	Tau isoform	Braak	Thal	Age	Sex	Fig.
TDP-43									
6	svPPA	AD (HpSp)/DLBD	0	3R + 4R	VI	5	60	M	
29	svPPA	AD	0	3R + 4R	VI	5	77	M	Fig. 7 A-C
30	agPPA	FTLD-TDP/HpScl (*GRN*)	A	--	0	0	64	M	Fig. 7 D-F; Fig. 8 D-I
31	FTD	FTLD-TDP/HpScl (*GRN*)	A	--	0	0	63	M	Fig. 7G-I
32	svPPA	FTLD-TDP	C	--	0	1	75	M	Fig. 7 J-L
33	agPPA	FTLD-TDP	C	--	I	1	65	M	
16	svPPA	PiD	0	3R	0	0	74	M	
α-Synuclein									
34	DLB	DLBD	0	--	0	0	60	M	
35	AD v DLB	DLBD	0	--	II-III	1	76	M	
36	MSA	MSA (SND & OPCA)	0	--	0	1	58	F	
Off-target									
1	Normal	Normal	0	--	0	0	81	F	Fig. 9 A,B
3	Normal	Normal	0	--	0	0	65	M	Fig. 9 C,D
19	PSP	PSP	0	4R	II-III	1	68	F	Fig. 9 E,F
6	svPPA	AD (HpSp)/DLBD	0	3R + 4R	VI	5	60	M	Fig. 9 G,H
36	MSA	MSA (SND & OPCA)	0	--	0	1	58	F	Fig. 9 I,J
30	agPPA	FTLD-TDP/HpScl (GRN)	A	--	0	0	64	M	Fig. 9 K,L
2	Normal	Normal	0	--	0	0	68	M	Fig. 9 M-P
31	FTD	FTLD-TDP/HpScl (GRN)	A	--	0	0	63	M	Fig. 9 Q-T
37	CBD	NBIA	0	3R + 4R	V	0	51	M	
38	VaP	Siderosis	0	--	0	0	59	F	

De-identified case numbers are referred to throughout the text and within Figs. TDP-43 type was performed using the recommended harmonized classification system [33]. Braak refers to neurofibrillary tangle stage [29] and Thal refers to amyloid phase [37]. Abbreviations: *svPPA* semantic variant of primary progressive aphasia, *agPPA* agrammatic variant of PPA, *FTD* frontotemporal dementia, *DLB* dementia with Lewy bodies, *AD* Alzheimer’s disease, *MSA* multiple systems atrophy, *PSP* progressive supranuclear palsy, *CBD* corticobasal degeneration, *VaP* vascular parkinsonism, *HpSp* hippocampal sparing AD, *FTLD-TDP* frontotemporal lobar degeneration of a TDP-43 etiology, *HpScl* hippocampal sclerosis, *GRN* progranulin mutation, *NBIA* neurodegeneration with brain iron accumulation, *3R* 3 repeat tau, *4R* 4 repeat tau, *M* male, and *F* female

### AV-1451 PET clinical patients

AV-1451 PET imaging cases were selected from the Mayo Clinic Study of Aging (MCSA) and Alzheimer’s Disease Research Center (ADRC) as described previously [34] based on anecdotal imaging findings and are included only for demonstration purposes of clinical variations. All participants provided written consent with approval of the Mayo Clinic Foundation and Olmsted Medical Center Institutional Review Boards. Cases were injected with 370 MBq (range 333-407 MBq) of F-18-AV1451prior to imaging and PET/CT imaging was performed as a 20-min PET acquisition between 80-100 min after injection. Images were optimized in grey scale to show brain contrasts within and between images.

### Tissue methods

Formalin-fixed and paraffin embedded tissue sections were cut at 5 μm thickness and used for both immunohistochemical and autoradiographic studies. Alternate sections were processed for immunohistochemistry and for AV-1451 autoradiography for best anatomically matching. Thioflavin-S microscopy was used to assign a Braak tangle stage and Thal amyloid phase, as previously described [15]. A DAKO Autostainer (Universal Staining System Carpinteria, California) was used to perform immunohistochemical staining with phospho-serine 409/410 TDP-43 (1:5000 mouse monoclonal; Cosmo Bio Co., LTD.), CP13 (1:1000 mouse monoclonal anti phospho-serine 202 tau, gift from Peter Davies), PHF-1 (1:1000 mouse monoclonal anti-phospho-serine 396/404 tau, gift from Peter Davies), and α-synuclein (1:3000 rabbit polyclonal anti-alpha-synuclein [35]). Antigen retrieval was performed by steaming sections in deionized water for thirty minutes; except for α-synuclein, for which sections were incubated in 98 % formic acid for 30 min and then steamed in deionized water for 30 min. Prussian blue stain was used to evaluate iron [36]. Braak NFT stage [29] and Thal amyloid phase [37] were assessed using thioflavin-S microscopy, as previously described [15].

### Radiosynthesis of AV-1451

The radiosynthesis of AV-1451 (7-(6-[^18^F] fluoropyridin-3-yl)-5H-pyrido [4,3-b] indole) was accomplished by nucleophilic fluorination of precursor AV1622 (5-(5-(tert-butoxycarbonyl)-5H-pyrido[4,3-b]indol-7-yl)-N,N,N-trimethylpyridin-2-aminium 4-methylbenzenesulfonate, AVID Radiopharmaceuticals, INC) in an automated TRACERlab FXN PRO synthetic module (GE Healthcare). The nucleophilic fluorination of AV 1622 (1.5 mg in 2.0 mL DMSO) was carried out at 110 °C for 5 min. The deprotection of amino group was carried out using 1 mL of 3 N HCl at 100 °C for 5 min followed by neutralization of the resultant solution with 7 mL of 0.5 N NaOH at 50 °C. The reaction mixture was passed through Oasis (HLB plus- from Waters) cartridge to remove DMSO and unreacted fluoride [^18^F^-^] before HPLC purification. The crude product was eluted from Oasis with 1.5 ml of acetonitrile and mixed with 3 mL of water before subjecting to HPLC purification. Product was purified on a semi-preparative HPLC using a reverse phase C-18 column Zorbax Eclipse XDB-C18, 9.4 × 250 mm, 5 μm (Retention Time = approximately 8 min) using the isocratic elution 60 % 10 mM ammonium acetate/water: 40 % acetonitrile at a 4 mL/min flow rate. The product was collected in 30 mL water and passed through a C-18 plus cartridge to concentrate. The dehydrated ethanol (1.2 ml) was used to elute the concentrated AV1451 and formulated in 0.9 % sodium chloride (9 ml) solution. The final product was transferred into a 30 mL sterile vial through a 0.22 μm sterilizing filter. The radiochemical purity, identity and specific activity of AV1451 was examined on an analytical HPLC system comprised of Agilent Eclipse XDB C18, 3.5 μm 75 × 4.6 mm column using 75 % water: 25 % acetonitrile: 0.1 % trifluoro acetic acid (TFA) as an eluent, flow rate 1.0 mL/min and UV 279 nm. AV1451 was found to be >99.0 % pure, and specific activity was found to be 7.743 ± 4.13 Ci/μmol (*n* = 155) at the end of synthesis.

### Autoradiographic methods

To correlate AV-1451 binding with PHF-1 and TDP-43 immunohistochemistry (IHC), autoradiography was performed on adjacent 5 μm sections from the 38 cases selected from our research brain bank. Autoradiography and blocking experiments were performed as previously described [38]. In short, each rehydrated section was incubated with 20 μCi AV-1451 in 500 μl PBS per section (Specific activity was 2.07 mCi/μmole at end of beam and 7.90 mCi/μmole at end of synthesis) at room temperature for 60 min. Each section was then washed in PBS for 1 min, in 70 % ethanol/PBS for 2 min, in 30 % ethanol/PBS for 1 min, and in PBS for 1 min to remove unbound AV-1451. During all rinsing steps, sections were rotated to ensure adequate removal of unbound AV-1451 was occurring. For determination of the nonspecific binding by displacement autoradiography, a solution of the nonradioactive reference compound AV-1451 in ethanol (3.8 mM) was added to a solution of [18 F] AV-1451 in phosphate buffer saline and diluted with PBS to give a final AV-1451 concentration of 2500 X Kd (36.5 uM) and a final [18 F] AV-1451 concentration of 2 MBq/ml. After incubation for 60 min at room temperature, unbound [18 F] AV-1451 was removed by washing the sections in PBS (1 min), 70 % ethanol/PBS (2 min), 30 % ethanol/PBS (1 min), PBS (1 min), and deionized distilled water (1 min). Tissue sections without any AV-1451 exposure and blank slides with [18 F] AV-1451 exposure were included as controls. After air-drying, each labeled section was exposed to a GE digital autoradiography film for 1 h and 16 h. Autoradiographic images were obtained using a GE FLA-7000 Phosphor Imaging Typhoon Scanner set at the highest sensitivity and at 25 μm resolution. Multiple (up to 3) autoradiographic procedures were performed on some samples on the same or nearby tissue section to exclude artifactual AV-1451 binding.

### Comparative assessment of AV-1451 binding

Visual comparison of autoradiographic AV-1451 uptake with immunohistochemistry was performed to assess AV-1451 binding. Autoradiographic findings were characterized as absent (no binding above background), minimal (binding slightly above background without focal areas of intense binding), moderate (binding greater than background with a few intense focal areas much greater than background) or strong (widespread focal and diffuse binding much greater than background). Immunohistochemical findings were reported as absent or present. Regions of adjacent and spatially correlated tissue were compared. Tissue regions evaluated included middle frontal, motor, inferior parietal and superior temporal cortices, hippocampus, amygdala, basal ganglia, subthalamic nucleus and midbrain, as well as white matter structures, including cerebral white matter, optic tract and fornix. The regions examined were chosen for their specific vulnerability to tau or other pathology depending on the disorder. A total of 98 distinct tissue samples were analyzed.

## Results

### AV-1451 PET imaging in patients with AD and non-AD neurodegenerative diseases

An example of antemortem AV-1451 PET imaging in a patient with clinically probable AD showed high uptake in temporoparietal and frontal cortices (Fig. 1 A-D). This patient also had positive amyloid imaging utilizing 11C-Pittsburgh compound B (PiB) PET. The patient’s 18 F-fludeoxyglucose (FDG) PET showed a pattern of hypometabolism similar to the pattern of high uptake with AV-1451. A patient with the agrammatic variant of PPA (agPPA) without apraxia of speech and predicted TDP-43 pathology had low-level AV-1451 uptake in the left temporal and left frontal lobes without uptake observed elsewhere in the cortex [39]. This patient was negative on PiB-PET and the FDG-PET showed hypometabolism predominantly on the left-side as is typical of agPPA (Fig. 1 E-H). A patient with the semantic variant of PPA (svPPA), also likely having TDP-43 pathology, had very mild AV-1451 uptake in the left temporal lobe without uptake observed elsewhere in the cortex. This patient was negative on PiB-PET and the FDG-PET showed hypometabolism predominantly on the anterior medial left temporal lobe as is typical of svPPA (Fig. 1 I-L). Antemortem AV-1451 PET imaging in a PSP syndrome patient revealed no specific brain uptake but did show uptake in the region of the substantia nigra that could be considered relatively nonspecific as it can be seen in most subjects. In *MAPT* mutation carriers, there was low uptake in a patient with *MAPT* S305N mutation who had advanced symptomatic disease and low uptake, much of which was in white matter, in another S305N mutation carrier who had moderate symptoms. A minimally symptomatic patient carrying the *MAPT* N279K mutation showed uptake in basal ganglia, but very low uptake in other areas. Nonspecific binding was observed in the meninges of the PSP syndrome patient, basal ganglia in the *MAPT* N279K patient and choroid plexus in the agPPA patient (Fig. 1 M-P). Observations in these and other patients guided selection of postmortem cases for AV-1451 autoradiography experiments.

#### Clinically relevant questions of interest

**Fig. 2:**
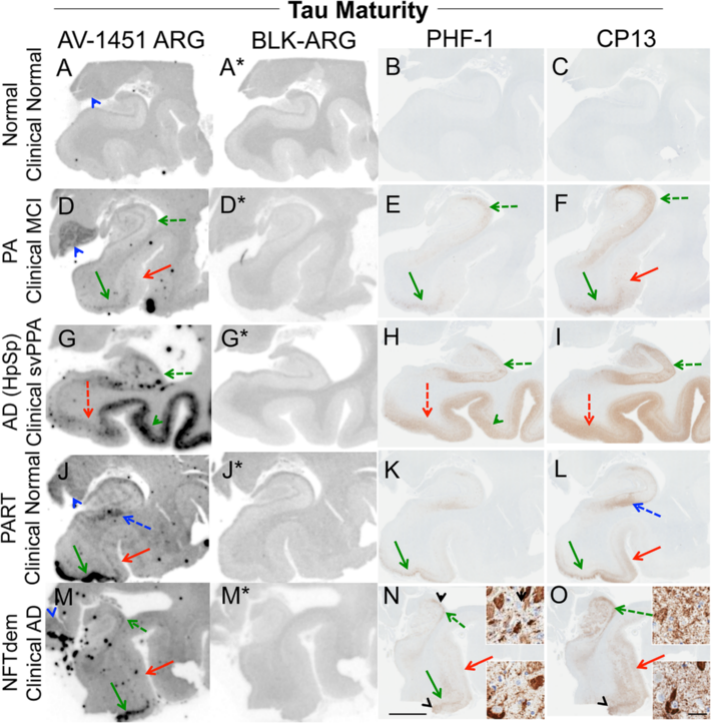
Correlative AV-1451 autoradiography (ARG) and immunohistochemical (IHC) findings assessing tau maturity, atypical AD and AD tauopathies. (**A**-**C**) Case 2 - Normal, (**D**-**F**) Case 5 – Pathological aging, (**G**-**I**) Case 6 – Hippocampal sparing (HpSp) Alzheimer’s disease (AD), (J-L) Case 10 – Primary Age-Related Tauopathy (PART), (M-O) Case 12 – Neurofibrillary tangle (NFT) predominant dementia. Columns from left to right show AV-1451 ARG, AV-1451 Blocked ARG (BLK-ARG, shown with “letter”*), PHF-1 and CP13 immunohistochemistry in the posterior hippocampal region in cases with various Braak neurofibrillary tangle stages (0-VI) and Thal amyloid phase (0-5). AV-1451 displaceable correspondence with IHC in the medial parahippocampal gyrus (*green arrows*) is strong with a trend for poorer AV-1451 signal correlation in the lateral parahippocampal gyrus (*red arrows*) seen especially relative to CP13 except in the hippocampal sparring AD case (**G**-**I**). The greatest AV-1451 and IHC concordance was observed in the occiptiotemporal gyrus in AD HpSp (case 6, green arrowhead). There is minimal-moderate AV-1451 signal in the hippocampal formation (CA1, *green dashed arrows*) that is modest relative to IHC in most samples, again especially as compared to CP13. In the Braak VI AD HpSp case (**G**-**I**, case 6), there is minimal AV-1451 signal in the medial parahippocampal gyrus (*red dashed arrows*) and minimal binding in the hippocampus (*green dashed arrows*) that is a mismatch to the PHF-1 and CP13. More muted AV-1451 signal was seen in the subiculum in PART (J-K, case 10, *blue dashed arrows*). Lateral geniculate AV-1451 diffuse, displaceable, minimal signal was seen in many cases (*blue arrowheads*) and some focal intense signal in some cases (case 10) that may represent vascular non-specific AV-1451 signal. Scale bar for all full size hippocampus at 5 mm, and for 20x zoomed insets at 25 μm. *Black arrowheads* indicate 20x inset locations

**Fig. 3:**
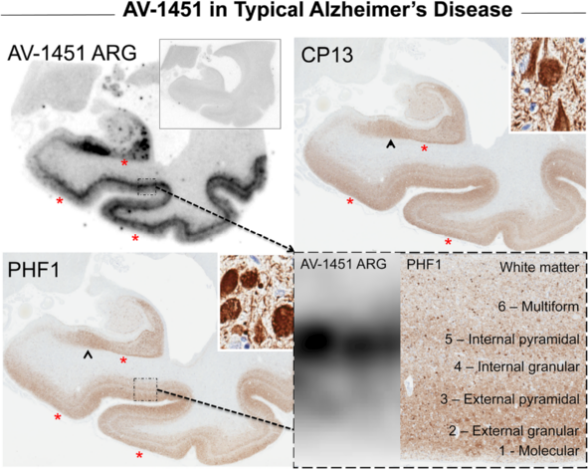
Correlative autoradiography (ARG) and immunohistochemical (IHC) findings for assessment of correlative binding in typical Alzheimer’s disease (AD, case 7). AV-1451 ARG (AV-1451 blocking shown in the left ARG inset), PHF-1 and CP13 immunohistochemistry in the posterior hippocampal region in a Braak VI, Thal 5 AD case. Tau pathology in the subiculum is shown in the upper right inset (*black arrowhead*) and shows very good signal on AV-1451 ARG, PHF-1 and CP13. However, the pattern of AV-1451 binding is similar but not identical to IHC with some focal discordance in many regions including Sommer’s sector of the hippocampus where CA1 and subiculum meet, the superficial layers of the parahippocampal and occipitotemporal gyri (*). Intense, linear AV-1451 signal in cortex matches best with the internal pyramidal cell layer (5) (*right lower inset*) while superficial layers (1-4) have more minimal-moderate AV-1451 signal where there is intense IHC positivity (*right lower inset* and *yellow arrow*). Scale bar for all full size hippocampus at 5 mm, and for 20x zoomed insets at 25 μm. *Black arrowheads* indicate 20x inset locations

**Fig. 4:**
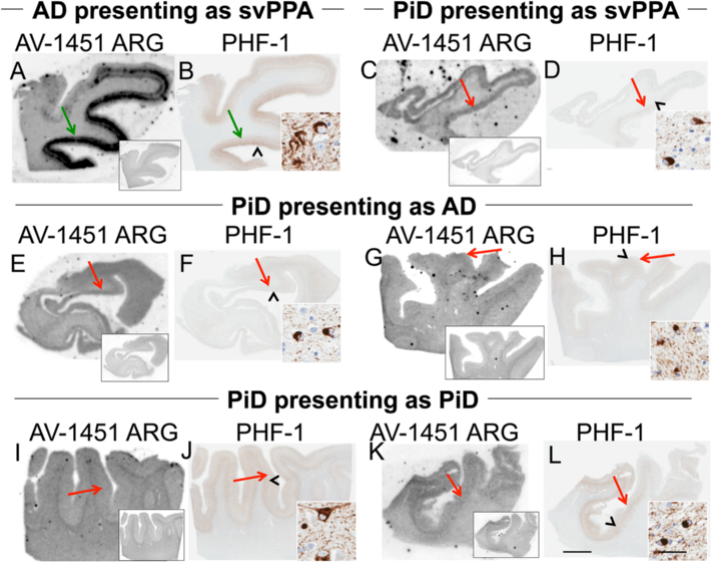
Correlative autoradiography (ARG) and immunohistochemical (IHC) findings for assessment of Pick’s disease (PiD, 3R Tau) binding. **a**, **b** Case 6 - AD for comparison (HpSp)/DLBD, **c**, **d** Case 14 - PiD, **e**-**h** Case 15 - PiD, **i**-**l** Case 16 – PiD. Shown from left to right AV-1451 ARG (AV-1451 blocking shown in insets) and PHF-1 in the **a**-**f** temporal cortex, **g**-**j** frontal cortex, and **k**-**l** parietal cortex for comparison. Moderate-severe AV-1451 displaceable binding in the AD case is shown (*green arrows*) corresponding with PHF-1. AV-1451 ARG displaceable signal is minimal and much weaker in the Pick’s cases than in the AD subject even in those with similar intensity of PHF-1 immunopositivity (**i**-**l**) to AD (*red arrows*). In some cases, loss of white matter-grey matter AV-1451 contrast may be the most notable feature of AV-1451 binding as compared to AV-1451 blocking studies (**g**, **i**). Scale bar for all full size cortical sections at 5 mm, and for 20x zoomed insets at 25 μm. *Black arrowheads* indicate 20x inset locations

**Fig. 5:**
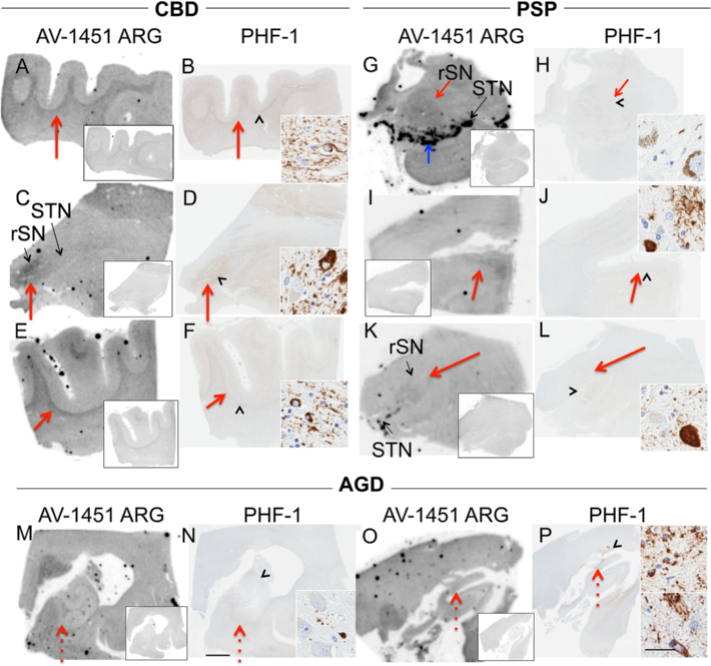
Correlative autoradiography (ARG) and immunohistochemical (IHC) findings for assessment of 4R Tau (CBD, PSP, and AGD) binding. **a**-**d** Case 17 – CBD (rostral substantia nigra (rSN), substantia nigra (STN)), **e**, **f** Case 18 - CBD, **g**, **h** Case 19 - PSP, **i**-**l** Case 20 - PSP, **m**, **n** Case 22 - AGD, **o**, **p** Case 23 – AGD. Shown from left to right AV-1451 ARG (AV-1451 blocking shown in the ARG insets), and PHF-1 IHC in the **a**, **b**, **e**, **f** frontal and **c**, **d** subthalamic regions in CBD cases; **g**, **h** midbrain in PSP cases; **i**, **j** frontal, **k**, **l** subthalamic; and **m**-**p** hippocampal regions in AGD cases. AV-1451 displaceable ARG binding corresponding to PHF-1 is often minimal and when present corresponds (*red arrows*) with the most dense areas of PHF-1 immunopositivity but is overall weaker than in AD subjects. Strong, non-tau related, ARG displaceable signal in the substantia nigra (*blue arrow*) may be cross-reacting with neuromelanin containing nigral neurons. Minimal displaceable ARG signal in rSN corresponds to PHF-1 immunopositivity (**g**, **k**, *red arrows*). No AV-1451 signal is seen that corresponds to PHF-1 positive AGD (*red dashed arrows*, **m**-**p**). Scale bar for all full size brain sections at 5 mm, and for 20x zoomed insets at 25 μm. *Arrowheads* indicate 20x inset locations

**Fig. 6:**
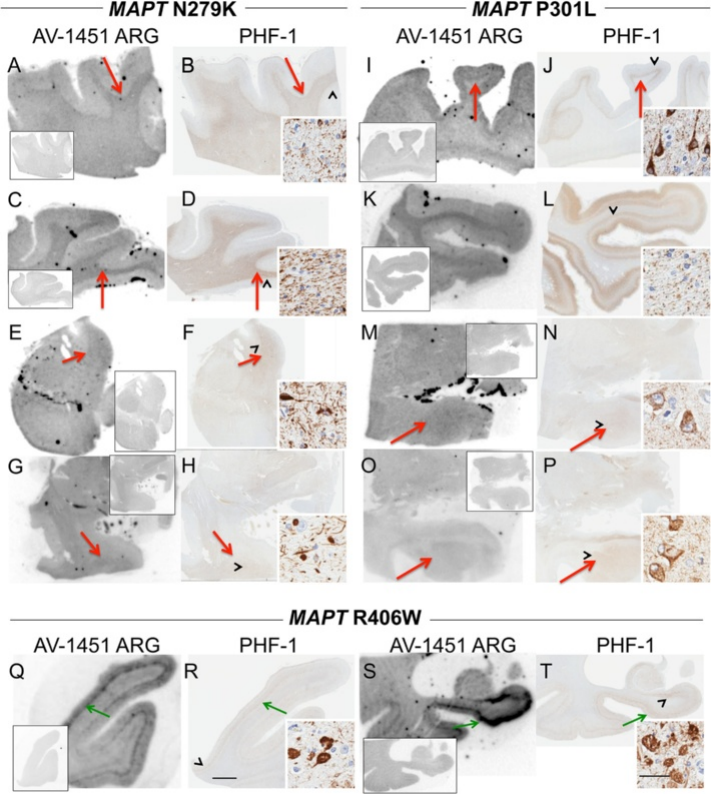
Correlative autoradiography (ARG) and immunohistochemical (IHC) findings for assessment of MAPT mutation (N297K and P3012L) tau binding. **a**-**f** Case 24 - FTDP-17 (MAPT N279K), **g**, **h** Case 25 - FTDP-17 (MAPT N279K), **k**-**n** Case 26 - FTDP-17 (MAPT P301L), **i**, **j**, **o**, **p** Case 27 - FTDP-17 (MAPT P301L), **q**-**t** Case 28 - FTDP-17 (MAPT R406W). Shown from left to right AV-1451 ARG (AV-1451 blocking shown in the ARG insets) and PHF-1 IHC in the **a**, **b**, **i**, **j** frontal, **c**, **d**, **k**, **l**, **q**, **r** temporal, **e**, **f** midbrain, **g**, **h**, **m**-**p** amygdala, and **s**, **t** hippocampus. AV-1451 displaceable ARG signal is minimal but co-localizes in most cases with the most dense areas of PHF-1 immunopositivity (*red arrows*). Conversely, the MAPT R406W case shows moderate-strong binding in cortical structures with a laminar pattern similar to that observed in AD (*green arrows*). Scale bar for all full size brain sections at 5 mm, and for 20x zoomed insets at 25 μm. *Arrowheads* indicate 20x inset locations

**Fig. 7:**
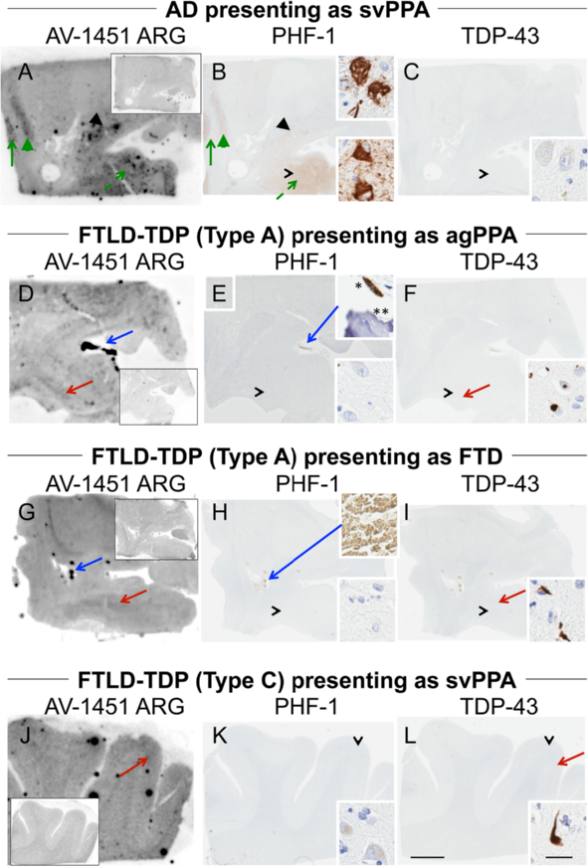
Correlative autoradiography (ARG) and immunohistochemical (IHC) findings for assessment of TDP-43 binding in cases with different phenotypic TDP-43-like presentations. **a**-**c** Case 29 – AD pathologically, **d**-**f** Case 30 - FTLD-TDP, **g**-**i** Case 31 - FTLD-TDP, **j**-**l** Case 32 - FTLD-TDP. Shown from left to right AV-1451 ARG (AV-1451 blocking shown in the ARG insets), and PHF-1 in the **a**-**i** amygdala and **j**-**l** frontal cortex. **a**-**c** A TDP-43-negative AD case presenting clinically as semantic variant of primary progressive aphasia (svPPA) shows strong ARG displaceable binding in insula (*green arrow*), claustrum (*green arrowhead*), and NBM (*black arrowhead* with inset, tangles) and amygdala (*black arrow* with IHC inset, tangles) with correspondence on PHF-1 IHC. **d**-**f** The amygdala region in a FTLD-TDP case presenting as an agrammatic variant of PPA (agPPA) with TDP-43 type A at autopsy, shows a negative PHF-1 and minimal diffuse ARG binding with a weak correspondence (*red arrows*) with TDP-43 pathology (TDP-43 inset, cytoplasmic and intranuclear inclusions). Strong off-target binding of the vasculature (*blue arrow*) appears to correspond with mineralization of the vessel and potentially with subpial melanin lentiform-shaped inclusion (upper inset). **g**, **i** Similarly, the amygdala of the FTLD-TDP case presenting as FTD with TDP-43 type A at autopsy was negative on PHF-1 and minimally positive on TDP-43. The ARG displaceable binding shows a weak correspondence (*red arrows*) with TDP-43 pathology (inset). Strong off-target binding of the vasculature (*blue arrow*) appears to correspond with cross-reactivity to red blood cells (H upper inset). **j**-**l** Frontal cortex in a FTLD-TDP case presenting clinically as semantic variant PPA (svPPA) with TDP-43 Type-C at autopsy was negative on PHF-1 and positive on TDP-43. ARG shows minimal displaceable binding in cortex that corresponds (*red arrows*) to TDP-43 pathology (inset, long thick dystrophic neurite). Scale bar for all full size brain sections at 5 mm, and for 20x zoomed insets at 25 μm. Black arrowheads indicate 20x inset locations

**Fig. 8:**
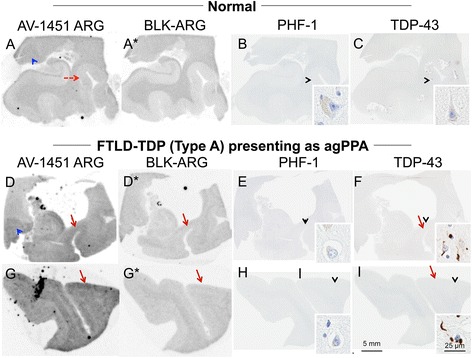
Correlative autoradiography (ARG) and immunohistochemical (IHC) findings for assessment of TDP-43 binding. (**a**-**c**) Case 2 - Normal, (**d**-**i**) Case 30 - FTLD-TDP. Shown from left to right AV-1451 ARG, AV-1451 Blocked ARG (BLK-ARG, shown with “letter”*), PHF-1 and TD-43 in the (**a**-**f**) posterior hippocampal and (**g**-**i**) superior temporal cortex. ARG images show no AV-1451 in the normal case (**a**-**c**) and minimal displaceable AV-1451 uptake in the (**d**) parahippocampal grey matter and (**g**) temporal lobe grey matter (*red arrows*) similar to regions of TDP-43 immunopositivity that is not seen in the normal brain (*red dashed arrow*, **a**). Minimal binding of AV-1451 is seen in the lateral geniculate nucleus (*blue arrowheads*) in both normal and TDP-43 positive cases is likely nonspecific. As an internal control, note that the grey matter parahippocampal AV-1451 signal in the normal case is absent (*red dashed arrow*) and the lateral geniculate (**a**) has greater signal—unlike the TDP case (**d**). Black arrowheads indicate 20x inset locations

**Fig. 9:**
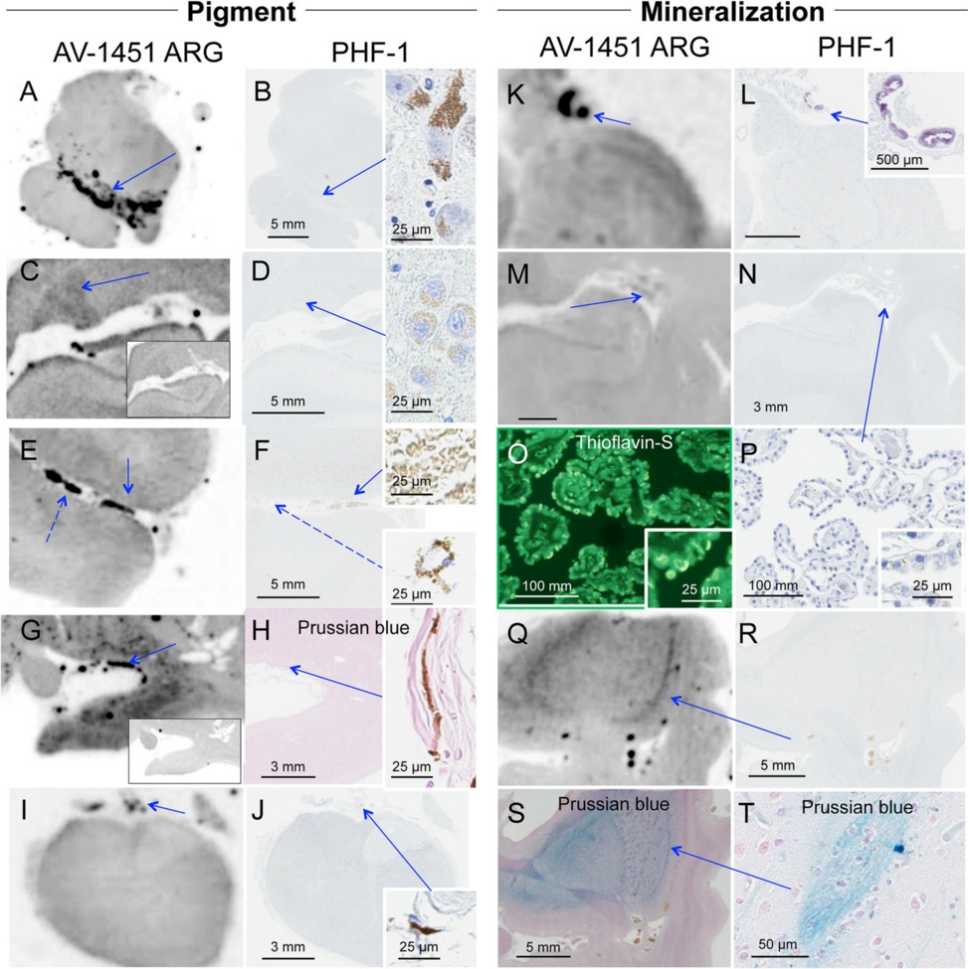
Correlative autoradiography (ARG) and immunohistochemical (IHC) examples of off-target AV-1451 binding. **a**, **b** Case 1 - Normal, **c**, **d** Case 3 - Normal, **e**, **f** Case 19 - PSP, **g**, **h** Case 6 - AD (HpSp), **i**, **j** Case 36 - MSA, **k**, **l** Case 30 - FTLD-TDP, **m**-**p** Case 2 - Normal, **q**-**t** Case 31 - FTLD-TDP. We present evidence for two major causes of off-target binding (*blue arrows*), **a**-**j** pigment-laden structures and **k**-**t** mineralization. Shown from left to right AV-1451 ARG and PHF-1 IHC (except where noted) are shown in the **a**, **b** midbrain, **c**, **d**, **k**-**p** posterior hippocampus, **e**, **f** superior temporal, **g**, **h**, **q**-**t** amygdala, and **i**, **j** pituitary. **a**, **b** AV-1451 signal is seen in the substantia nigra in all cases in the study (*blue arrow*); **c**, **d** lateral geniculate displaceable (**c**, inset) in most cases likely owing to the abundance of pigment-containing lipofuscin-filled neurons (inset); **e**, **f** vascular structures on some cases in vessels with cross-reactive red blood cells (top inset); **g**, **h** subpial coverings in some cases with strong displaceable (**g**, inset) binding observed in proximity to melanin-containing structures that were also found on non-antibody based stains (**h**, Prussian blue, inset); **i**, **j** moderate AV-1451 binding in the pituitary section is also found to correspond to subpial melanin. **k**, **l** Strong, focal AV-1451 binding was observed in the choroid plexus and found to correspond to mineralization of the vessel (inset). **m**, **n** ARG of choroid plexus revealed variable binding with some cases showing little-to-no binding. PHF-1 staining did not reveal tau-positive structures in choroid. Closer inspection of choroid with (**o**) thioflavin-S fluorescent microscopy revealed abundant Biondi in older brains, which corresponded to (**p**) pigment-containing structures on zoomed PHF-1 slides. **s**, **t** Variable involvement of basal ganglia was noted, but was not found to correspond with tau pathology. **s** A digitally-modified Prussian blue (iron stain) revealed a similar pattern underlying moderate-strong ARG binding. Curiously, pencil fibers were often noted to be Prussian blue positive. Scale bar for all full size and zoomed brain sections are noted within each image. *Black arrowheads* indicate 20x inset locations

The subject characteristics in the postmortem study are found in Tables 1 and 2 (see Additional file 1 for all AV-1451 scoring). A total of 38 cases were examined. Results for the several clinically relevant questions of interest were as follows:

1. Tau maturity, Atypical AD and AD
  AV-1451 binding corresponded moderately well to tau deposition in cases with Braak Stages from 0 to VI in patients with various clinical presentations and developmental tau stages of atypical AD. (Fig. 2 A-I). AV-1451 binding corresponded to tau deposition in a clinical svPPA patient (with hippocampal sparing AD at autopsy [30]) (Fig. 2 G-I). Interestingly, in this hippocampal sparing AD, with Braak Stage VI, only minimal to focally moderate AV-1451 binding was observed in the hippocampus. In contrast, the occipitotemporal and inferior temporal cortices showed strong AV-1451 binding, which corresponded better with localization of PHF-1 immunopositive pretangles and mature tangles. Cases that had Braak Stages I-IV but lacked amyloid pathology were neuropathologically classified along the PART- tangle predominant dementia spectrum [20]. PART cases showed moderate-to-strong binding in the parahippocampal cortex that corresponded to severe PHF-1 staining (in 2 of 3 cases), while there was minimal -moderate binding in CA1-subiculum of the hippocampus that corresponded to mild-to-moderate PHF-1 staining (example in Fig. 2 J-L). Tangle predominant dementia cases had relatively variable minimal-to-strong AV-1451 binding in the parahippocampal cortex (example in Fig. 2 M-O). In fact, despite numerous NFTs, including many extracellular (“ghost”) NFTs in the hippocampus in some cases, there was minimal AV-1451 binding in the hippocampus in tangle predominant dementia. Typical AD cases (Fig. 3) showed strong binding throughout the parahippocampal and temporal cortices, as well as in the CA1/subiculum of the hippocampus, which corresponded to severe tau pathology. At higher resolution, AV-1451 showed inconsistent binding to regions that had extensive tau neuritic pathology on the IHC-adjacent section, most notably in superficial cortical layers. The characteristic laminar distribution of tau pathology that is distinctly visible on the autoradiographic image corresponded to cortical layers with NFT in pyramidal cell layers rather than granule cell layers of the cortex (Fig. 3, right lower inset). AV-1451 binding generally corresponded better with PHF-1 than CP13 across the range of AD-type neurofibrillary pathology in PART, tangle predominant dementia and AD as seen in Fig. 2.
2. Isoform variations of tau and AV-1451
  Strong, specific AV-1451 binding, like that observed in AD, was not seen in any of the cases with 3R (i.e., PiD) (Fig. 4) or 4R tauopathies (i.e., PSP and CBD) (Fig. 5). PiD autopsy cases presenting as svPPA, AD dementia, and behavioral variant FTD clinical syndromes demonstrated absent or minimal AV-1451 binding that when observed, corresponded to PHF-1 immunoreactivity in the adjacent section. AV-1451 binding in CBD (Fig. 5 a-f red arrows), PSP (Fig. 5 g-l red arrows), and AGD (Fig. 5 m-p) was absent or minimal, but when detected corresponded to the location of PHF-1 immunoreactivity in the adjacent section. Absent-to-minimal AV-1451 binding was seen in FTDP-17 cases with N279K and P301L mutations and again corresponded to the location of PHF-1 immunoreactivity in the adjacent section (Fig. 6 a-p). When observed, minimal AV-1451 binding (red arrows) in all of these cases corresponded to cortical white matter or grey matter areas of severe PHF-1 immunoreactivity. AV-1451 binding in FTDP-17 cases was absent-to-minimal in brainstem and amygdala. Strong, specific AV-1451 binding, however, was observed to correspond with PHF-1 immunoreactivity in the R406W mutation case (Fig. 6 q-t). Moderate-to-strong midbrain binding was seen in every case, which will be described in the section of “off-target” binding.
3. TDP-43 and AV-1451
  We assessed AV-1451 autoradiographic binding in FTLD-TDP in selected cases with variable clinical presentations. Amygdala tissue sections sampled at the level of the anterior commissure showed strong AV-1451 binding in pathologic AD, including those who presented with clinical svPPA (*n* = 2), particularly in the amygdala, basal forebrain, nucleus basalis, claustrum, and insula (example, Fig. 7 a-c). AV-1451 binding corresponded well with PHF-1 immunohistochemical staining in the adjacent tissue section. In comparison, FTLD-TDP cases (*n* = 4) showed neither moderate nor strong AV-1451 binding in any of the cases in areas with TDP-43 immunoreactivity on adjacent sections. The AV-1451 binding was however mildly positive in some FTLD-TDP cases showing minimal AV-1451 binding that was always less than that observed in AD. In two of the three FTLD-TDP cases there was minimal AV-1451 binding in gray matter regions in the frontal (Fig. 7 j-l), parahippocampal and temporal cortices (Fig. 8 D-I). Minimal AV-1451 binding was likewise seen in a pathologic PiD case that presented clinically with a svPPA phenotype (Fig. 4 c, d).
4. AV-1451 and α-synuclein
  The two dementia with LBD (DLBD) cases, which presented clinically as DLBD and AD dementia, showed no AV-1451 binding in the amygdala, basal forebrain or basal ganglia, areas with significant α-synuclein immunoreactivity on the adjacent sections. The MSA case presented clinically as atypical parkinsonism with autonomic dysfunction and had no significant AV-1451 binding. The midbrain in all three cases with α-synuclein pathology had no α-synuclein-specific binding.
5. AV-1451 off-target binding
  AV-1451 binding in midbrain was prominent in all relevant tissue samples regardless of disease type and was much more intense than any corresponding immunoreactivity to PHF-1 on the adjacent sections (Fig. 9 a, b). Many posterior hippocampal sections showed minimal-moderate binding of the lateral geniculate nucleus, which corresponded to a higher density of lipofuscin-rich neurons (Fig. 9 c, d). Binding of AV-1451 co-localized with nonspecific vascular PHF-1 immunoreactivity in multiple brain regions of both control and disease cases. This nonspecific binding may correspond to AV-1451 binding to a subpopulation of red blood cells (Fig. 9 e, f). Subpial AV-1451 binding was often detected in areas that had PHF-1-negative melanin-containing structures (Fig. 5 e-h). In one case, AV-1451 binding was noted in the pituitary and this also corresponded to melanin-containing structures (Fig. 9 i, j). Binding to choroid plexus was infrequent, but was more apparent in older cases and co-localized in one case to dystrophic calcification in the choroid plexus (Fig. 9 k, l). The presence of Biondi bodies did not appear to correspond with stronger binding in choroid plexus (Fig. 9 m-p). Other areas of non-specific AV-1451 uptake were observed in the meninges and scalp (Fig. 1) and basal ganglia (Fig. 9 s, t). Minor displaceable binding was observed in normal white matter and grey matter (Fig. 2A, A*).

## Discussion

This work demonstrates that AV-1451 binding on autoradiography corresponds relatively well to tau pathology demonstrated by immunohistochemistry, with preferential binding to AD-type tau pathology, although the relationship is surprisingly complex. AV-1451 binding is absent or minimal in non-AD primary tauopathies and TDP-43 proteinopathies, and absent in α-synucleinopathies. We also provide evidence for several types of AV-1451 “off-target” binding, which we hypothesize to either be of a pigment-based or mineralization-based etiology.

A few previous studies have described AV-1451 binding characteristics in brain tissue [19, 38, 40], but these prior reports did not include many novel observations that our study includes. First, our study includes IHC capable of detecting differences in AV-1451 binding to tau to predominant forms of early and late neurofibrillary tangle maturity, where CP13 assesses earlier tau pathology and PHF-1 assesses more advanced tangle pathology. We found differences in the association of AV-1451 with tau maturity using both PHF-1 and CP13 immunohistochemical staining. This difference implies that AV-1451 is specific to particular hyperphosphorylated regions or related conformations and therefore has variable binding depending on the maturity of neurofibrillary tangle pathology. CP13 is an antibody to anti phospho-serine 202 tau that phosphorylates at an early time in tau hyperphosphorylation relative to PHF-1 (phospho-serine 396/404 specific) [41]. A demonstration of this effect is observed in Fig. 2 (D,E,F) where more CP13 positive regions are seen relative to PHF-1 and AV-1451 correlates better with PHF-1 findings or a presumably a later hyperphosphorylation time point. A related novel finding is the variable layering of AV-1451 in the grey matter that was often found in AD cases (Fig. 3), which has not been described previously. These findings have important implications to the sensitivity of AV-1451 at different stages of tau maturity and are described in more detail below. Second, we provide evidence for the first time describing AV-1451 binding in PART and tangle predominant dementia. Both neuropathologies may underlie medial temporal lobe changes measured by structural MRI or PET studies. Our study provides the first foundation related to these two pathologies and putative labeling by tau imaging as compared to AD. Third, TDP-43 associated AV-1451 binding is demonstrated as a sporadic finding (similar to the findings of Sander, et al,[38]) and additional patient examples are provided to demonstrate potential tauopathies underlying clinical phenotypes of FTD (Fig. 4) and are therefore additionally possible explanations for AV-1451 positive scans in FTD patients. Fourth, while ref 35 does describe some examples “off target’ binding, the present paper shows additional examples that are not described in the prior literature that include vascular, choroid plexus, basal ganglia, pituitary, and subpial AV-1451 binding.

AV-1451 autoradiography showed marked binding to multiple cortical and limbic regions in AD, where tau pathology is composed of relatively equimolar 3R and 4R tau [42]. The AV-1451 binding was strong and diffusely positive in cases with high Braak NFT Stages as expected and as previously described [19]. However, we observed different AV-1451 binding patterns within the regions when comparing two phospho-tau antibodies (PHF-1 and CP13) with different profiles on immunoreactivity with tau positive structures in AD that describe early (CP13) and later (PHF-1) tau development. The PHF-1 profile is as follows: neuritic pathology < pretangles < mature tangles > extracellular tangles (with pretangles and extracellular tangles being similarly labeled); while CP13 (labels neuritic pathology ≈ pretangles > mature tangles > > extracellular tangles. The findings depicted in Fig. 2 suggest that early tau deposition, as best shown by CP13, is not represented well in AV-1451 autoradiography (i.e., medial parahippocampal gyrus (red dashed arrows), lateral parahippocampal gyrus (red arrows) and subiculum and CA1 (green dashed arrows) regions, Fig. 2 D-F, J-L). PHF-1 and AV-1451 moderate correspondence is consistent at each successive Braak NFT stage. Although tangle predominant dementia typically has marked medial temporal lobe AD-type tau pathology with Braak NFT stages III or IV, hippocampal neuronal loss is much higher than would be expected in early AD or the mild spectrum of PART [4, 43]. Tau pathology in tangle predominant dementia is primarily composed of extracellular 3R tau positive “ghost” tangles [44]. This shift from a 3R + 4R tau ratio to an overrepresentation of 3R could explain the relatively minimal AV-1451 binding we observed in tangle predominant dementia (Fig. 2 M-O). On the recent paper by Marquie et al. [19], ghost tangle (extracellular) binding of AV-1451 is described on nuclear emulsion autoradiography in AD-tau but the finding is not well demonstrated as no individual IHC and AV1451 comparison images are provided and the technique is therefore unable to confirm our findings. Tangle predominant dementia cases may be the extreme form of PART [20]. There is much controversy surrounding the nature of the tau pathology in PART and whether PART is actually an early stage of AD [45, 46]. Mild PART cases (Fig. 2 J-L) provide supportive evidence that a critical mass of NFTs may be needed for strong AV-1451 binding, as the medial parahippocampal gyrus region has many more NFTs (PHF-1 inset) than the subiculum and also has much stronger AV-1451 binding. As PART is defined by lack of amyloid pathology or minimal amyloid (Thal amyloid phase ≤ 2; possible PART) [37], multi-modal approaches using amyloid PET will be useful – especially as evidence supports Thal amyloid phase ≤ 2 roughly equates to the threshold in established using our PIB data processing pipeline [15]. Knowledge of amyloid burden is of particular importance in AD cases with atypical distribution of tau pathology, as is evidenced by case #6, (Fig. 2 G-I). This case of advanced AD presented as a non-amnestic, svPPA and was not suspected of having AD. AV-1451 binding showed moderate-to-strong binding in cortex, but minimal-moderate binding in hippocampus (green dashed arrow) as the hippocampus was relatively spared of NFT [30]. Of note, off-target binding in the lateral geniculate nucleus was observed in this case, which could confound lower resolution clinical AV-1451 PET scan findings as a result of AV-1451 uptake in a region near the hippocampus.

An interesting pattern of AV-1451 binding was seen in cortical gray matter of advanced AD cases, such as in Fig. 3. AV-1451 was most prominent in deeper cortical layers, corresponding to pyramidal cell layers, and reduced in upper cortical layers, corresponding to molecular layer and granular cell layers. A similar layering effect can be seen in Marquie et al. [19], (Fig. 2, row A, left box) but was not described in that work. This pattern of AV-1451 binding that we observed contrasted with results of tau immunohistochemistry where many tau positive neurites were detected in upper cortical layers. The association of AV-1451 binding to NFTs more than neurites may explain preferential binding to layers with pyramidal neurons as well as the observation of focal, intense AV-1451 binding in CA1 and the subiculum of the hippocampus, reflecting areas with densely packed NFTs. The findings suggest a preference of AV-1451 binding to NFT and less to neurites, but it is not clear if or why such a preference would exist. It could be due to a higher molecular abundance of tau associated with cell bodies compared to neurites. There could be tau conformational differences or differences in filament organization in neurites compared to NFT that correlated with AV-1451 binding. Implications related to maturity of tau may be postulated as well. There are related reports indicating differences in timing of tau pathology with initial pathologic changes in AD occurring in different layers of the entorhinal/parahippocampal cortex [47, 48]. These preferential binding mechanisms remain however speculative and further work is needed to elucidate the pathologic mechanisms of these findings.

We observed a wide variation in AV-1451 binding in non-AD neurodegenerative tauopathies. Prior work with other tau-PET probes has shown that THK-523 [49] does not bind to non-AD tau and PBB-3 [50] does bind to tau from non-AD tauopathies namely Pick’s disease, PSP and CBD. Marquie et al [19], present that AV-1451 does not bind to non-AD tauopathies or TDP-43 but Sanders, et al, have data that suggest that AV-1451 does bind to non-AD tauopathies (greatest in PiD and FTDP-17) and additionally to FTLD-TDP Types A and C (1/3 cases for each type). Our findings for AV-1451 align best with those of Sanders, et al.. We have not tested the other tau-PET probes but it is clear that differences in tau specificity of various probes will make clinical utilization complicated. Different methods of investigation such as fluourescence with PBB-3 [50] make direct comparison between these reports problematic. Head-to-head testing of the different probes would be helpful. The intensity of AV-1451 uptake that we see in different tauopathies could be a reflection of the different tau isoforms or structural differences in tau filaments. Higuchi and colleagues have reported that AD and several other diseases (tangle predominant dementia, Down syndrome, Guam-Parkinson-dementia complex, atypical CJD with NFTs, Niemann-Pick disease type C, and FTDP-17 with R406W *MAPT* mutation) have morphologically similar neurofibrillary structures composed of 95 % PHFs and minimal straight filaments (SFs) [51]. In contrast, PiD and PSP have neurofibrillary structures that contain more SFs and fewer PHFs. Morphologically different ribbon-like tau filaments are typical of FTDP-17 and CBD; these filaments are wider and have irregular periodicity of 90 to 130 nm [51]. In addition, while all tau isoforms are present in AD, 3R isoforms predominate in PiD and 4R isoforms predominate in CBD and PSP. Either morphological or isoform differences could be implicated in the different intensity of AV-1451 binding in these disorders. The tendency of AV-1451 to show greater intensity of localization in AD than PiD, PSP or CBD would suggest that AV-1451 has greater affinity for PHF-rich neurofibrillary structures as opposed to SF-rich structures (PiD and PSP), as recently suggested [19]. Our data adds to these findings by demonstrating strong binding in the R406W *MAPT* mutation case, which was also seen by Sander et al., corresponding to moderate-severe PHF-1 immunoreactive tau pathology. Filament specificity is supported by our observations of poor correspondence between ARG and IHC in N279K mutation carriers (prevalence of mixed twisted ribbon and straight filaments) and P301L mutation carriers (predominantly narrow twisted filaments), compared to R406W mutation carriers (mix of paired helical filaments and straight filaments). The different isoforms may also play a role in the different specificity. The strength of AV-1451 binding with respect to tau isoforms that preferential accumulate in various neurodegenerative disorders was as follows: 3R + 4R tau (e.g., AD) > 3R tau (e.g., Pick disease) or 4R tau and also matches the pattern seen by Sander et al. Our findings suggest that disorders with tau pathology have sufficient differences with respect to binding to PET ligands that they may need to be tailored to particular tau species.

We found no consistent evidence for correspondence of AV-1451 with TDP-43 pathology. In the FTLD-TDP cases, the amygdala and frontal lobe tissue showed no AV-1451 binding although these areas had TDP-43 pathology on adjacent sections. On the other hand, we did find some AV-1451 binding greater than background in regions of TDP-43 pathology in two of the three FTLD-TDP cases in frontal, parahippocampal and temporal cortices. These results may represent genuine, weak co-localization with TDP-43, although we cannot confirm this, but there may be other unexplained pathologic causes. One potential explanation relates to severe degeneration of subjacent white matter in many cases of FTLD-TDP, which may lack background or basal-level radiotracer uptake, thus enhancing the contrast of signal between gray and white matter. Data from clinically imaged FTLD patients with high likelihood of TDP-43 pathology, such as those with svPPA, will be necessary to evaluate the frequency of this finding, with eventual confirmation depending upon postmortem studies. There have been anecdotal reports (unpublished) of AV-1451 PET uptake in svPPA cases. We demonstrate that cases with the clinical phenotype of svPPA may have AV-1451 PET positive scans and tau pathology due to unsuspected AD pathology and draw attention to the consideration of this and other tau pathologies as potential causes of such AV-1451 findings in svPPA [52, 53]. In any case, these data suggest that there is minimal-to-no AV-1451 binding in TDP-43 proteinopathies and that any observed binding is at levels much less than that in AD. The other major group of non-tauopathies is associated with α-synuclein deposition. We found no evidence for AV-1451 binding to regions with dense α-synuclein pathology on adjacent sections in either MSA or LBD.

Off-target AV-1451 binding patterns in the basal ganglia corresponding with Prussian blue staining, (Fig. 9 q, r), led us to postulate that AV-1451 may associate with iron. We also observed high AV-1451 binding in the substantia nigra. Thus, we performed two observational experiments to test this hypothesis: 1) the substantia nigra, another region known to accumulate iron, and 2) a case with brain iron accumulation who was genetically predisposed to abnormally accumulate iron. The strong binding of AV-1451 to the substantia nigra in all cases, including cases with no tau pathology, suggests a potential binding to iron. Iron binds to high affinity sites of neuromelanin to form stable complexes that protect against iron overload [54]. Experimental iron overload of neuromelanin results in low affinity iron to exacerbate neurotoxicity through oxidative processes. Intrinsic melanin binding may be an alternative explanation for substantia nigra AV-1451 signal as postulated by others [19]. Although significant Prussian blue staining was observed in the globus pallidus of the NBIA case, we did not observe a matching pattern of AV-1451 binding. Minimal AV-1451 binding in the red nucleus was seen corresponding to PHF-1 (Fig. 5 g, h, red arrows), which reflects a region susceptible to neuronal loss in CBD and PSP and related tau pathology. This binding was minimal in comparison to AV-1451 binding in the substantia nigra, which could make specific, tau-involved AV1451 signal identification in regions adjacent to the substantia nigra challenging.

Other “off-target” AV-1451 binding sites as seen in Fig. 9 have implications that vary with respect to minor non-specific binding to interfering with interpretation. Potentially the most clinically relevant “off-target” binding regions are the substantia nigra (for confounding of red nucleus tau binding in PSP), the lateral geniculate binding in hippocampal Braak tangle staging, subpial melanin-containing structures, and the choroid plexus or vascular structures when closely associated with the hippocampus or other Braak-important regions. “Off-target” binding in the pituitary and binding to scalp or calcification may be less important. “Off-target” binding may provide clues regarding the molecular target of the AV-1451 ligand. To this end, we sought to bin the “off-target” examples based on their similarities. Within in the pigment-related category, we included neuromelanin-containing neurons, lipofuscin-filled lateral geniculate neurons, and subpial melanin-containing structures. There is evidence to suggest that melanin interacts with amyloidin (functional amyloid) [55], suggesting that perhaps neuromelanin may be recognized based on its structure and not necessarily protein sequence. AV-1451 binding to calcifications in choroid plexus and in areas known to accumulate iron was binned into a mineralization-related category. Contrary to clinical findings suggesting strong AV-1451 signal in choroid, our autopsy study revealed variations – perhaps suggesting there is a dynamic process that we are not able to assess in the postmortem tissue. Inspection of the choroid plexus using thioflavin-S microscopy reveals fluorescent Biondi ring-like tangles [56]. We observed Biondi bodies regardless of the strength of AV-1451 binding, but interestingly they have been found to contain melanin at the ultrastructural level.

## Conclusions

In conclusion, we demonstrate the relative specificity of AV-1451 to 3R + 4R tau over other isoforms of tau. We demonstrate minimal binding in association with TDP-43 pathologically involved regions and no association with α-synuclein pathologies. Our findings suggest that multi-modal tau PET and amyloid PET studies may be useful for differentiating atypical AD with non-amnestic presentations from other proteinopathies. These data suggest that patients with strongly positive AV-1451 scans and svPPA could indeed have tau pathology (probably underlying AD-tau) while patients with minimally positive AV-1451 scans more likely have TDP pathology. AV-1451 was found to bind preferentially to NFT compared to neurites. Moreover, AV-1451 had a predilection for mature tangles over extracellular “ghost” tangles. These observations, along with our findings of variable AV-1451 binding in tangle predominant dementia, suggest that variable signal of AV-1451 may be seen with tau disease progression due to variations in tau location and isoforms. A limitation of this work includes the small number of individual cases for each disease. The lack of clinical PET AV-1451 imaging in these cases prevents direct comparisons of the minimal areas of binding and off-target binding with PET imaging which would be needed to make more accurate correlations of the findings.

## Ethics, consent, and permissions

All participants provided written consent with approval of the Mayo Clinic Foundation and Olmsted Medical Center Institutional Review Boards.

## Consent to publish

All participants provided written consent to allow publication of anonymized participant data.

## Additional file

Additional file 1: Detailed Patient Demographics and Results of ARG Scoring. (PDF 74 kb)
