# Supplementary material for: An autoradiographic evaluation of AV-1451 Tau PET in dementia
Source: Acta Neuropathol Commun. 2016 Jun 13;4:58. doi: 10.1186/s40478-016-0315-6 (PMC4906968; doi:10.1186/s40478-016-0315-6)
Supplement: Additional file 1: — Detailed Patient Demographics and Results of ARG Scoring. (PDF 74 kb) [file 40478_2016_315_MOESM1_ESM.pdf]

Case ID	ClinicalDx	PathDx	TDP type	Tauopathy	Braak	Thal	Age	Case ID	Sex	Figure
1	Normal	Normal	0	--	0	0	81	1	F	Fig 9 A,B
2	Normal	Normal	0	--	0	0	68	2	M	Fig 2 A-C; Fig 8, A-C; Fig 9 M,N,O,P
3	Normal	Normal	0	--	0	0	65	3	M	Fig 9 C,D
4	Normal	PA	0	3R+4R	III	3	94	4	F	
5	MCI	PA	0	3R+4R	III	2	88	5	F	Fig 2 D-F
6	PPA-S	AD (HpSp)/DLBD	0	3R+4R	VI	5	60	6	M	Fig 2, G-I; Fig 4 A,B; Fig 9 G,H
7	AD	AD (Typical)/DLBD	0	3R+4R	VI	5	78	7	F	Fig 3 A-D
8	PSP	PART/VaD	0	3R+4R	III-IV	0	57	8	F	
9	Normal	PART	0	3R+4R	III-IV	0	89	9	F	
10	Normal	PART	0	3R+4R	IV	0	82	10	F	Fig 2 J-L
11	MCI	NFT dementia	0	3R+4R	III	0	81	11	F	
12	AD	NFT dementia/VaD	0	3R+4R	IV	0	82	12	M	Fig 2 M-O
13	AD	NFT dementia/AGD/VaD	B	3R+4R	III-IV	0	80	13	F	
14	PPA-S	Pick's	0	3R	0	0	74	14	M	Fig 4 C,D
15	AD	Pick's (lobar atrophy)	0	3R	I	1	70	15	M	Fig 4 E-H
16	PiD	Pick's (lobar atrophy)	0	3R	0	0	72	16	M	Fig 4 I-L
17	PPA-G	CBD/PA	0	4R	I-II	2	67	17	M	Fig 5 A-D
18	AD	CBD/AGD	0	4R	II	0	67	18	M	Fig 5 E,F
19	PPA-G	PSP/AGD/SRT/TSA	0	4R	II	0	80	19	M	Fig 5 I-L; Fig 9 E,F
20	PSP	PSP	0	4R	II-III	1	68	20	F	Fig 5 G-H
21	PSP	PSP	0	4R	I	0	65	21	M	
22	MCI	AGD	0	4R	III	0	90	22	M	Fig 5 M,N
23	Normal	AGD	0	4R	III	1	91	23	M	Fig 5 O-P
24	PPND	FTDP-17 (PPND; MAPT N279K)	0	4R	0	0	49	24	M	Fig 6 A-F
25	PPND	FTDP-17 (PPND; MAPT N279K)	0	4R	0	0	50	25	F	Fig 6 G,H
26	FTD	FTDP-17 (MAPT P301L)	0	4R	0	0	53	26	M	Fig 6 K-N
27	FTD, familia	FTDP-17 (MAPT P301L)	0	4R	I-II	0	52	27	M	Fig 6 O,P
28	FTD	FTDP-17 (MAPT R406W)	--	3R+4R	5	0	66	28	F	Fig 6 Q-T
29	PPA-S	AD (Typical v HpSp)/CAA	0	3R+4R	VI	5	77	29	M	Fig 7 A-C
30	PNFA	FTLD-U/HpScl (GRN)	A	--	0	0	64	30	M	Fig 7 D-H; Fig 8 D-I; Fig 9 K,L
31	FTD	FTLD-U/HpScl/NII (GRN)	A	--	0	0	63	31	M	Fig 7 G-I; Fig 9 Q-T
32	PPA-S	FTLD-U	C	--	0	1	75	32	M	Fig 7 J-L
33	PNFA	FTLD-U	C	--	I	1	65	33	M	
34	DLB	DLBD	0	--	0	0	60	34	M	
35	AD v DLB	DLBD	0	--	II-III	1	76	35	M	
36	MSA	MSA (SND & OPCA)/SC	0	--	0	1	58	36	F	Fig 9 I,J
37	CBD	NBIA (PSD)/NFT/LBD	0	3R+4R	V	0	51	37	M	
38	VaD	Siderosis	0	--	0	0	59	38	F	

Case ID	ARG score region 1	ARG score region 2	ARG score region 3	ARG score region 4	ARG score region 6
1	Hippocampus; absent	Midbrain; absent			
2	Hippocampus; absent				
3	Amygdala; absent	Hippocampus; absent	Midbrain; absent		
4	Hippocampus; moderate				
5	Hippocampus; minimal				
6	Amygdala; moderate	Temporal; strong	Hippocampus; strong		
7	Amygdala; moderate	Parietal; strong	Visual; strong	Hippocampus; strong	Midbrain; absent
8	Hippocampus; moderate				
9	Hippocampus; minimal				
10	Hippocampus; moderate				
11	Hippocampus; strong				
12	Hippocampus; moderate				
13	Hippocampus; absent				
14	Amygdala; minimal	Temporal; minimal	Frontal; absent		
15	Frontal; minimal	Parietal; minimal			
16	Amygdala; absent	Temporal; minimal	Frontal; minimal-moderate	Parietal; minimal	
17	Amygdala; minimal	Frontal; minimal-moderate	Motor; absent	Midbrain; absent	Subthalamic; minimal
18	Amygdala; absent	Frontal; minimal	Motor; minimal	Midbrain; absent	Subthalamic; minimal
19	Amygdala; absent	Frontal; minimal	Motor; absent	Midbrain; minimal	Subthalamic; minimal
20	Frontal; absent	Motor; minimal	Midbrain; minimal	Subthalamic; minimal	
21	Frontal; minimal	Motor; absent	Subthalamic; minimal		
22	Hippocampus; absent				
23	Hippocampus; absent				
24	Amygdala; absent	Temporal; minimal	Frontal; minimal	Midbrain; absent	
25	Amygdala; minimal	Temporal; absent	Frontal; absent	Midbrain; absent	
26	Amygdala; absent	Temporal; minimal	Frontal; minimal	Midbrain; absent	
27	Amygdala; minimal	Temporal; minimal	Frontal; minimal	Midbrain; absent	
28	Hippocampus; strong	Temporal; strong			
29	Amygdala; strong	Temporal; strong	Frontal; strong	Parietal; strong	Visual; moderate
30	Amygdala; absent	Temporal; minimal	Frontal; minimal	Hippocampus; minimal	
31	Amygdala; minimal	Temporal; minimal	Frontal; minimal		
32	Amygdala; minimal	Temporal; minimal	Frontal; minimal		
33	Amygdala; absent	Frontal; minimal			
34	Frontal; absent	Midbrain; absent			
35	Amygdala; absent	Midbrain; absent			
36	Midbrain; strong	Pituitary; moderate			
37	Hippocampus; absent				
38	Frontal; absent				

Case ID	ARG off target scores
1	substantia nigra; strong
2	lateral geniculate: minimal
3	substantia nigra; strong; lateral geniculate: minimal
4	lateral geniculate: minimal
5	lateral geniculate: minimal
6	ependymal
7	substantia nigra; strong; lateral geniculate: minimal
8	
9	strong; lateral geniculate: minimal
10	lateral geniculate: minimal
11	lateral geniculate: minimal
12	ependymal strong; lateral geniculate: minimal
13	ependymal strong; lateral geniculate: minimal
14	basal ganglia, vessels
15	
16	
17	substantia nigra; strong
18	substantia nigra; strong
19	ependymal and substantia nigra; strong
20	substantia nigra; strong
21	
22	vessels
23	
24	ependymal and substantia nigra; strong
25	ependymal and substantia nigra; strong
26	ependymal and substantia nigra; strong
27	ependymal and substantia nigra; strong
28	
29	basal ganglia, vessels
30	
31	basal ganglia, vessels
32	
33	basal ganglia, ependymal
34	substantia nigra; strong
35	substantia nigra; strong
36	ependymal in pituitary; substantia nigra strong
37	nucleus basalis: strong
38	vessels

Information:

Title: An Autoradiographic Evaluation of AV-1451 Tau PET in Dementia

Journal: Acta Neuropathologica

Author: Val J. Lowe, MD
Department of Radiology, CH 1-285
Mayo Clinic
200 First Street SW
Rochester, MN 55905 USA
Telephone 507 284-4104
Email: vlowe@mayo.edu
